# A statistical framework for consolidating "sibling" probe sets for Affymetrix GeneChip data

**DOI:** 10.1186/1471-2164-9-188

**Published:** 2008-04-24

**Authors:** Hua Li, Dongxiao Zhu, Malcolm Cook

**Affiliations:** 1 Bioinformatics Center, Stowers Institute for Medical Research, 1000 E 50th St, Kansas City, MO 64110, USA; 2 Department of Computer Science, University of New Orleans, New Orleans, LA 70148, USA; 3 Research Institute for Children, Children's Hospital, New Orleans, LA 70118, USA

## Abstract

**Background:**

Affymetrix GeneChip typically contains multiple probe sets per gene, defined as sibling probe sets in this study. These probe sets may or may not behave similar across treatments. The most appropriate way of consolidating sibling probe sets suitable for analysis is an open problem. We propose the Analysis of Variance (ANOVA) framework to decide which sibling probe sets can be consolidated.

**Results:**

The ANOVA model allows us to separate the sibling probe sets into two types: those behave similarly across treatments and those behave differently across treatments. We found that consolidation of sibling probe sets of the former type results in large increase in the number of differentially expressed genes under various statistical criteria. The approach to selecting sibling probe sets suitable for consolidating is implemented in R language and freely available from .

**Conclusion:**

Our ANOVA analysis of sibling probe sets provides a statistical framework for selecting sibling probe sets for consolidation. Consolidating sibling probe sets by pooling data from each greatly improves the estimates of a gene expression level and results in identification of more biologically relevant genes. Sibling probe sets that do not qualify for consolidation may represent annotation errors or other artifacts, or may correspond to differentially processed transcripts of the same gene that require further analysis.

## Background

**Figure 1:**
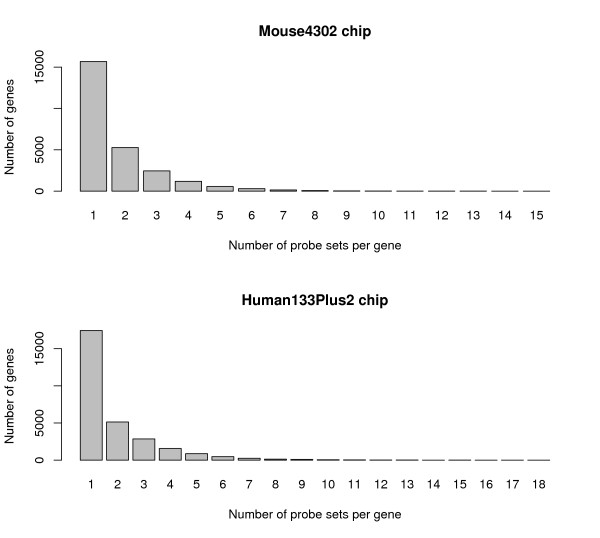
**The distribution of sibling probe set numbers per gene in Affymetrix Human and Mouse chips**. The figure describes the distribution of the probe set numbers per gene on the Affymetrix mouse moe4302 chip and human hgu133Plus2. About 40% of genes are presented by multiple probe sets, half of which are represented by three or more sibling probe sets, and some genes have even more than ten sibling probe sets.

Affymetrix GeneChip is one of the most popular platforms for profiling gene expression at the genome scale. It has been used for detecting differentially expressed genes [1-4], discovering disease markers [5], discovering functionally related genes, and clustering genome-wide expression patterns [6-9]. A single gene may be represented by multiple probe sets on a GeneChip. For example, in the mouse moe4302 chip, there are 45, 101 probe sets corresponding to 25, 724 distinct genes, and 40% of all genes are represented by multiple probe sets, called "sibling probe sets" throughout this paper. For these 40% of genes, almost half of them are represented by more than two probe sets on the chip, and some genes even have more than ten probe sets. Similarly in the human hgu133plus2 chip, the total of 28, 919 genes are represented by 54, 675 probe sets on the chip (Fig. 1).

According to Affymetrix, there are three primary reasons for designing sibling probe sets for the same gene: first, some cDNAs may be thought to come from different loci at the time of chip design, but later genome annotation maps them to the same gene; second, some probe sets turn out to cross-hybridize in an unpredictable manner, and additional probe sets with better specificity are designed for the same gene; third, probe sets specific to RNA variants, such as products of alternative splicing, or highly similar gene family or transcripts with different polyA sites, have been designed on purpose. Correspondingly, Affymetrix probe set name suffixes try to indicate these design purposes, such as probe sets with "s" and "x" suffixes are thought to be prone to cross-hybridization, and probe sets with an "a" suffix represent alternative splicing variants. However, two independent studies showed that different expression scores of sibling probe sets are not due to the inclusion of these suboptimal probe sets, and there is lack of evidence showing that these suboptimal probe sets performed worse than "better designed" probe sets [10,11]. Clearly the sibling probe sets problem must be tackled in analyzing Affymetrix microarray data, but the existing strategies have been very different.

Naive approaches to sibling probe sets are either to treat them in the same way as different genes [12] or to arbitrarily choose one sibling probe set as the representative of the gene and ignore the other sets [13,14,10]. For example, Jordan et al proposed to select the probe set with the highest expression value among the siblings [14], whereas Liao and Zhang [10] randomly picked one sibling probe set for their analysis. All these approaches solve the problem by discarding data in an arbitrary manner. There does not seem to be a systematic guideline for consolidating sibling probe sets. In the effort of remapping the probes to probe sets for creating a custom Chip Definition File (CDF), Dai et al [15] defined one gene mapping one probe set to avoid "redundant probe sets" in gene chip analysis. It has been shown that these updated probe set definitions provide both better precision and accuracy in probe set expression estimates compared to the original Affymetrix definition of hgu133a chip [16].

Elbez et al studied how well sibling probe sets measure the same gene expression on Affymetrix hgu133a GeneChip [11]. Using correlation statistics, they defined two groups of probe set pairs – pairs that are highly correlated and pairs that are not. They derived an empirical rule for Affymetrix hgu133a GeneChip that highly correlated sibling probe sets should be consolidated and others should not be. However, their approach suffers from the following limitations. First, they did not study multiple probe sets (more than 2) correlation, as about 18% of genes on the mouse chip have 3 or more sibling probe sets (Fig. 1). Second, only informative pairs (probes sets showing changes in transcription among different measurements) are included in their analysis, whereas the pairs that show no difference in expression are left alone, which possibly introduces some bias in results. Recently, Stalteri and Harrison published a case study using a mouse gene "Surf4" and determined that some sibling probe sets on the mouse moe430a array with inconsistent measures were to detect alternative splicing (poly(A) sites) or errors [17].

It seems appropriate to consolidate sibling probe sets that behave similarly, since they are more likely to be hidden replicates of the expression values of the same target gene. In contrast, sibling probe sets showing inconsistent expression values may represent real biological phenomena, or perhaps stem from annotation errors or other artifacts, and should not be consolidated in either case. In this work, we propose a statistical method for consolidating the sibling probe sets in the context of detecting differentially expressed genes over two or more physiological/genetic conditions. We cast the problem of automatic determination of the sibling probe set type in the ANOVA framework, in which the differential expression between sibling probe sets, treatments and their mutual influence are simultaneously inspected in a two-way ANOVA model (Eq. 1) or it's extension with block effect (Eq. 2) and test whether their interaction is significant. Insignificant interaction effect indicates that sibling probe sets are more likely to behave similarly and provides evidence for consolidation. This approach is referred as the per-gene approach throughout the paper.

We compare our approach to the two existing approaches: the per-probeset approach and the custom CDF approach. The per-probeset approach treats all sibling probe sets as distinct genes and is widely used in the literature. The custom CDF approach uses the redefined probe sets by assembling all probes mapping to the same gene to one probe set based on the genome database. There are usually multiple versions of custom CDFs for one platform due to multiple genome databases. For example, the UniGene custom CDF maps to the UniGene database. Using three publicly available Affymetrix datasets [18-20], we show that the per-gene approach is able to call more biologically relevant genes than the two other approaches.

## Results

### The Statistical Framework for Consolidating Sibling Probe Sets

**Figure 2:**
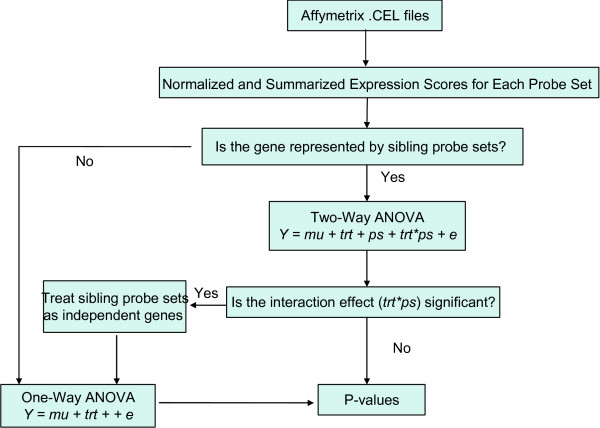
**The algorithm flowchart**. The figure demonstrates the outline of identification and consolidation of qualified sibling probe sets based on statistical tests. We are interested in studying the differentially expressed genes across treatments. The analysis starts from properly normalized and summarized expression scores for each probe set. For genes that are represented by multiple probe sets (sibling probe sets), insignificant interaction effect (trt*ps) between treatment (trt) and probesets (ps) suggests consolidating sibling probe sets and P-values of the treatment effect are obtained from the two-way ANOVA model. For the gene corresponding to a single probe set and those probe sets that are not eligible for consolidating, i.e. significant interaction effect (trt*ps), *P*-values of the treatment effect are reported from the one-way ANOVA model. Then *P*-values are combined as a final result for screening differentially expressed genes across treatments.

**Figure 3:**
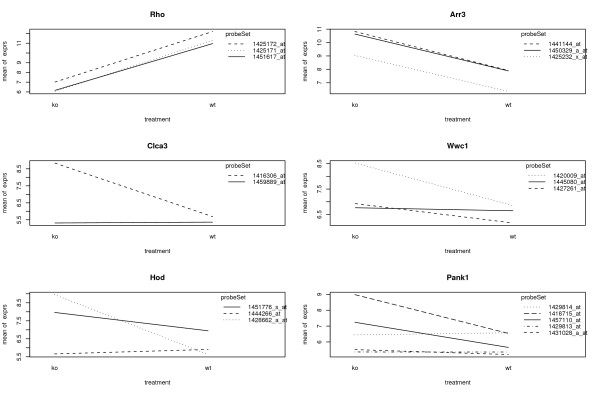
**Examples of sibling probe sets need to be consolidated (top row) and otherwise (middle and bottom rows)**. Examples of sibling probe sets need to be consolidated (top row) and otherwise (middle and bottom rows). In the top row, the differential expression of genes "Rho"and "Arr3" between wild type and treatment (Nrl knockout) does not change over sibling probe sets (raw *P*-values for interaction effect are 0.68, 0.89 respectively). In the middle and bottom rows, the differential expression between wild type and treatment for sibling probe sets have either different magnitude or reversed trend. The former is translated into a need for consolidation but not the later.

The outline of automatic identification and consolidation of qualified sibling probe sets based on statistically supported evidence is shown in Fig. 2. We start our analysis from properly normalized and summarized expression scores for each probe set, e.g. RMA score [21], GCRMA score [22] or Model-Based Expression Index (MBEI) [23]. We ask whether the differential expression over treatments among sibling probe sets follow the same trend or not in a two-way ANOVA model, which includes treatment (*τ*), probe set (*ψ*), as well as their interaction effect (*τψ*). Non-significant interaction effect indicates that the sibling probe sets have the same trend of differential expression over treatments. As shown in the top row of the Fig. 3, several probe sets show similar expression profile (slopes) between wild type and treatment (knock-out) and will be consolidated. Consequently, the *P*-value of treatment effect should be reported based on the two-way ANOVA model (Eq. 1) since it accounts for all measures from sibling probe sets for the same gene. Significant interaction effect indicates that the expression profiles from the probe sets are different in slopes shown in the middle and bottom rows of Fig. 3. These sibling probe sets are more appropriately treated as independent probe sets although they share same gene symbol. For independent probe sets or single probe sets, we compare differential expression over treatments using one-way ANOVA model (Eq. 3). In this case, *P*-values of treatment effect are reported from one-way ANOVA model.

It is often seen that the microarray experiment involves paired samples, for example, a pair of treatment and control samples are from the same individual. For these experiments, we add a block factor to the existing one-way (Eq. 4) and two-way ANOVA model (Eq. 2) to take into account the correspondence relationship between each pair.

**Figure 4:**
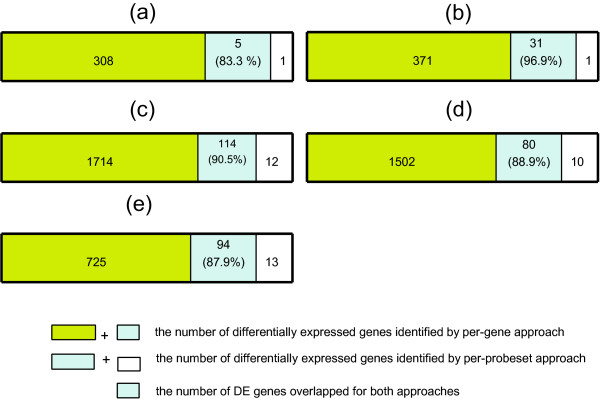
**Per-gene approach dominates per-probeset approach**. The percentages of overlapped genes detected by both approaches are shown in the overlapped areas. The numbers of differentially expressed genes identified by the per-gene approach and the per-probeset approach are: a. Data Set1, 313 vs. 6, overlap: 83.3% b. Data Set2, 402 vs. 32, overlap: 96.9% c. Data Set3, MBEI pre-processing, 1828 vs. 126, overlap: 90.5% d. Data Set3, RMA pre-processing, 1592 vs. 90, overlap: 88.9% e. Data Set3, GCRMA pre-processing, 832 vs. 107, overlap: 87.9%.

We compared the proposed per-gene approach with the existing per-probeset approach and custom CDF approach on the Affymetrix platforms moe4302 and hgu133plus2. We used different custom CDFs annotated from UniGene, ensEMBL gene and Entrez genome databases. Under the same FDR cut-off as well as *P*-value cut-off, we say that approach A **dominates **approach B if the gene list generated by A is much longer than that generated by B, and vast majority of the list B falls into the list A (Fig. 4). The approach that identifies the gene list enriched with experimentally relevant GO terms indicates better performance.

### Example 1: Discriminative Analysis over Treatment and Control

In the first example we compared the per-gene, the per-probeset and the custom CDF approaches by screening differentially expressed genes between Nrl knockout and wild type mouse at developmental stage Postnatal day 10 (P10) [18]. Nrl is the Maf-family transcription factor and the key regulator of photoreceptor differentiation in mammals. Nrl knockout causes slow but progressive vision loss in mammals [24]. We used RMA [21] to get the expression value for each probe set.

For 15, 632 genes that are represented by a single probe set on moe4302 GeneChip (Fig. 1), we performed one-way ANOVA with both equal variance and unequal variance assumption. Correspondingly for the 10, 049 genes that are represented by multiple sibling probe sets, we performed two-way ANOVA analysis with interaction between the two fixed effects *τ *and *φ *(Eq. 1). Specifically, we model probe set (*φ*) and treatment (*τ*) (Wild Type vs. Nrl-ko) as two factors as well as their interaction (whether differential expression changes over probe sets or *vice versa*). There are 62 sibling probe set genes whose interaction terms were called significant at False Discovery Rate (FDR, Benjamini-Hochberg (BH) Procedure [25]) no larger than 1%. It means that the differential expression over wild type and Nrl-ko conditions is dependent on the sibling probes sets or *vice versa*. For this reason we treated the 255 probe sets mapping to these 62 genes as individual probe sets, followed by fitting the one-way ANOVA model with treatment effect only (Eq. 3, Fig. 2). Finally, raw *P*-values of the treatment effect were combined from the full two-way ANOVA and the one-way ANOVA. The number of hypotheses tests reduced from 45, 101 in the per-probeset approach to 25, 917 = 15, 675 + 10, 049 - 62 + 255 in the per-gene approach.

Given an FDR cutoff (e.g. 5%), the per-gene approach calls more true differentially expressed genes between wild type and Nrl-knockout under different statistical assumptions and dominates over the per-probeset approach. Assuming the wild type and Nrl-ko populations have unequal variance, the per-probeset approach called only six probe sets, the UniGene custom CDF approach calls only 16 genes, the ensEMBL gene custom CDF approach calls only ten genes to be significantly differentially expressed with FDR smaller or equal to 5% while the per-gene approach called 313 genes at the same FDR cut-off (Table 1, Additional File 1). Five out of six probe sets determined to be differentially expressed by the per-probeset approach (Nrl, Rho, 4921511K06Rik, Gnb1, Lrp4) are included in the 313 probe set list (Fig. 4a, overlap 83.3%, Additional File 1, highlighted in yellow). For example, Nrl acts synergistically with Crx to regulate the transcription of Rho [24], and Gnb1 serves as a membrane receptor for signal transduction cascade regulating Rho [26]. We also examined 308 additional probe sets discovered at the same FDR by the per-gene approach and found many well-characterized genes that are directly or indirectly regulated by Nrl (see Additional File 1, highlighted in red). For example, Pde6b and Pde6h are directly regulated by Nrl [27]. Rp1h and Arr3 are also responsive to Nrl knockout [24,28]. Seven out of 16 genes called by the UniGene custom CDF approach and seven out of ten genes called by the ensEMBL gene custom CDF approach are included in the 313 gene list called by the per-gene approach. However, Rho, Gnb1, Arr3, and Rp1h, which are known to be regulated by Nrl, are not detected by neither the UniGene nor the ensEMBL gene custom CDF approaches.

**Table 1:** Performance comparisons in terms of numbers of differentially expressed genes.

	Mult-test Algorithm	Unequal Variance	Equal Variance
Per-Gene	Bonferroni (.05)	39	45
	FDR-BH (.05)	313	434
	FDR-BY (.05)	63	84
	RawP cut-off (6.5e-05)	124	151
Per-ProbeSet	Bonferroni (.05)	2	6
	FDR-BH (.05)	6	59
	FDR-BY (.05)	1	4
	RawP cut-off (6.5e-05)	6	59
customCDF-UniGene	Bonferroni (.05)	3	18
	FDR-BH (.05)	16	87
	FDR-BY (.05)	1	8
	RawP cut-off (6.5e-05)	18	40
customCDF-ensEMBLgene	Bonferroni (.05)	6	10
	FDR-BH (.05)	10	103
	FDR-BY (.05)	1	10
	RawP cut-off (6.5e-05)	18	59

Comparison of the per-gene approach, the per-probeset approach, the UniGene custom CDF approach, and the ensEMBL gene custom CDF approach in terms of screening differentially expressed genes between wild type and Nrl knockout.

The analyses under the assumption of equal variance and using the other multi-test correction methods such as Bonferroni, raw *P*-values cut-off and FDR under general dependency (Benjamini-Yeuketieli Procedure, BY) [29] follow the same trend (Table 1).

### Example 2: Cancer Gene Markers Identification using Paired Samples

In the second example, we compared the three approaches by screening differentially expressed genes between paired normal and thyroid cancer tissues as potential molecular markers on the Affymetrix hgu133plus2 Array. The data set (GSE3678) contains gene expression profiles of seven Papillary Thyroid Carcinoma (PTC) samples compared to seven paired normal samples. GCRMA [22] was used to normalize and summarize expression score for each probe set in each tissue sample. Since this data set is different from the mouse chip data analysis because of paired data, we reported *P*-values from the extended two-way ANOVA model with patient as a block effect (Eq. 2) for the genes that its representative multiple probe sets are consolidated (insignificant interaction effect between probeset and treatment). For the independent probe set or the single probe set, we reported *P*-values from the extended one-way ANOVA model with patient as a block effect (Eq. 4). Note that the latter analysis corresponds to the familiar paired t-test of treatment effect.

Controlling FDR at the level of 0.01 using "BH" procedure, the per-gene approach and the per-probeset approach call 402 and 32 differentially expressed genes between normal and PTC samples respectively, while the UniGene custom CDF approach made 24 significant calls and the ensEMBL gene custom CDF approach made 25 significant calls. It consistently shows that the per-gene approach dominates the per-probeset approach in that 31 out of 32 probe sets (Fig. 4b) called by the per-probeset approach were also called by the per-gene approach. 23 out of 24 genes that are identified by the UniGene custom CDF approach and 22 out of 25 genes that are identified by the ensEMBL gene custom CDF approach are also identified by the per-gene approach. Using other multiple tests correction procedures follows the same trend (see Additional File 2).

We then compared our approach with the per-probeset and the custom CDF approach using two strata of biological knowledge: cancer related gene ontology terms and true positive genes that are individually validated using traditional biochemical and genetics approaches. According to five cancer related GO terms, the per-gene approach outperforms both per-probeset and custom CDF approaches in light of enrichment *P*-values (Table 2). Huang et al [30] collected 7 well-studied genes in their publication as true positive PTC marker. DPP6, DPP4 (liver dipeptidyl peptidase IV), SFTPB, CHI3L1, MUC1 are known over-expressed genes in PTC samples. TPO and DIO2 are genes involved in thyroid metabolism. TPO is playing central roles in thyroid gland function, and DIO2 activates thyroid hormone by converting the prohormone thyroxine (T4) by outer ring deiodination (ORD) to bioactive 3,3',5-triiodothyronine (T3). At FDR cutoff 1%, our approach is able to pick out all seven genes while none was picked out by either the per-probeset or the custom CDF approach. It provides compelling evidence that our approach dominates over competitors and is capable of identifying more biologically relevant genes.

**Table 2:** Comparison in terms of cancer functional categories.

	PerGene	PerPS	customCDF-UniGene	customCDF-ensEMBLgene
Apoptosis	0.07567058	0.648036408	NA	NA
Cell Growth	0.091233514	0.219401388	NA	NA
Cell Differentiation	0.000731174	0.918393769	NA	NA
Cell Adhesion	0.000642603	0.078656064	0.042166	NA
Blood Vessel Development	0.004064254	NA	NA	NA

Comparison of the per-gene approach, the per-probeset approach, the UniGene custom CDF approach, and the ensEMBL gene custom CDF approach in terms of enrichment of informative GO terms.

### Example 3: Spermatogonial Stem Cell Self-Renewal Gene Markers Identification

In order to determine whether the per-gene approach consistently outperforms the per-probeset and the custom CDF approach under varied experiment conditions such as multiple treatment, normalization and summarization methods, we further compared three approaches on a third data set. The third microarray data set (GSE4799) profiled gene expression over five time-points before and after GDNF/GFR*α*1 replacement with a total of 15 samples. For this data set, we used GCRMA [22], RMA [21] and MBEI [23] pre-processing methods for Affymetrix CDF and three version of custom CDFs (UniGene, EntrezGene, and ensEMBL gene). Similar to our previous analysis, we reported *P*-values from (Eq. 3) or (Eq. 1) depending on whether the interaction effect is significant.

Comparing to the per-probeset approach, we, once again, found the per-gene approach dominates the per-probeset approach using FDR cutoff of 0.01 (BH procedure) for all three pre-processing methods (Fig. 4c–e). Similar to the second example, we compared the three approaches in terms of associated important GO terms such as "Chromatin remodeling", "Cell Differentiation" and "Regulation of Cell Growth" (Table 3). Although the results using different normalization methods are slightly different for all three approaches, the per-gene approach consistently shows the significant enrichments for all three GO terms, suggesting it is the best approach to identify genes that are associated with stem cell self-renewal process.

**Table 3:** Comparison in terms of stem cell self-renewal functional categories.

		Chromatin Remodeling	Cell Differentiation	Regulation of Cell Growth
MBEI	Per-gene	0.0575	**0.0013**	0.0512
	Per-ps	NA	0.0715	**0.0345**
	customCDF-UniGene	NA	0.0821	0.3545
	customCDF-EntrezGene	NA	0.2937	0.5050
	customCDF-ensEMBLGene	NA	0.4075	0.4724
RMA	Per-gene	0.0651	**0.0021**	**0.0013**
	Per-ps	NA	**0.0161**	0.4006
	customCDF-UniGene	NA	0.0731	0.3448
	customCDF-EntrezGene	NA	0.6577	0.0272
	customCDF-ensEMBLGene	NA	0.4075	0.0256
GCRMA	Per-gene	**0.0297**	**0.0060**	**2.52E-05**
	Per-ps	NA	0.1064	**0.0173**
	customCDF-UniGene	NA	**0.0446**	0.2237
	customCDF-EntrezGene	NA	0.7695	0.1004
	customCDF-ensEMBLGene	NA	0.5947	0.1209

Comparison of the per-gene approach, the per-probeset approach and the custom CDF approaches in terms of enrichment of informative GO terms.

## Discussion and Conclusion

We have demonstrated the advantages of consolidating sibling probe sets whenever possible in the context of detecting differential expression using popular Affymetrix moe4302 and hgu133plus2 platforms. Consolidating sibling probe sets is determined automatically through statistical test of probe set by treatment interaction effect in the two-way ANOVA model. It improves the analysis in two ways. First, pooling data from sibling probe sets improves the estimation of mean and variance of the observed gene expression level so that the significance of differential expression (*P*-value) is more accurately estimated. Second, pooling enhances the power of statistical tests, because it reduces the number of simultaneously hypothesis tests by consolidating the redundant sibling probe sets into one probe set. Like all the other approaches, the per-gene approach is also susceptible to the gene annotation. In cases that Affymetrix annotation linked distinct genes that happen to have a similar expression pattern in the given experiment, this approach will fail to separate these genes.

Formulating sibling probe sets consolidating rule is still an open problem. Elbez et al identified the problem of current Affymetrix probe set mapping is due to inaccurate genome annotation through analyzing the so-called "bad pairs" [11], and Dai et al derived the consolidating rule *externally *using customized CDF in a bottom-up fashion, i.e., using the most updated genome annotation from diverse databases to redefine the mapping of probes to probe sets so as to consolidate sibling probe sets [15]. The set of *post hoc *assembled solutions are useful and have been shown to provide better estimation of gene expression [16].

We addressed the same issue using a data-driven approach, that is, our approach does not rely on any databases, but rather formulate a consolidating rule *internally *using expression data of sibling probe sets.

We want to emphasize that we do not anticipate giving a universal recommendation to always consolidate some sibling pairs of probe sets. To the contrary, our approach provides a method to consolidate sibling probe sets whenever applicable, and consolidation is only based on the observed data in a particular experiment. We have no intention to predict the consolidation rule in a new data sets based on the one derived from previous analyzed data sets. As illustrated in our Additional File 3 and data from Elbez et al [11], expression values of sibling probe sets might show a high correlation in one experiment by not in another. However, causes of probe set pairs showing a high correlation in one data set, but a low correlation in another are not well studied.

Our framework may affect subsequent analysis such as clustering and networking. For example, in both gene clustering and networking, the focus is often on a small subset of differentially expressed genes. Without consolidating sibling probe sets, the per-probeset approach often retains redundant probe sets of the same gene, which is not only problematic for network and clustering visualization and interpretation, but also substantially lowers the statistical power of the biological discovery. In gene set enrichment analysis using enrichment score [31], the expression value of the gene could be denoted by the mean of expression values of multiple probe sets that mapping to the same gene if these multiple probe sets are consolidated based on statistical tests.

Another important feature of the per-gene approach to rank differentially expressed genes is: the well-characterized genes (functions may still remain elusive) are more enriched in the top ranked list produced by the per-gene approach than by the per-probeset approach. One possible explanation is that Affymetrix designs sibling probe sets mostly for the well-characterized genes. Consolidating these sibling probe sets wherever applicable will substantially increase the sample size for more reliable detecting the differential expressions for these genes. The per-gene approach is particularly useful for less well-annotated genomes for which the enrichment of well-characterized genes in the top ranked list would markedly facilitate our understanding the underlying biological process.

## Methods

### Data Sets

The first Affymetrix data set we used was generated by Akimoto et al [18] using Affymetrix mouse moe4302 chip. The data was downloaded from the Gene Expression Omnibus (GEO) database using accession number GSE4051. We focused on identifying differentially expressed genes at developmental maturity stage P10 with 4 replicates in both wild type and Nrl-ko conditions. We chose to compare the differentially expressed genes between wild type and knockout at developmental stage P10, as it reflects the popular experimental design in microarray analysis for comparing two conditions. The P10 is chosen because it is the starting point of the mature state of photoreceptor differentiation.

The second Affymetrix data set we used was generated by Reyes et al [19] using Affymetrix human hgu133plus2 chip. The data was downloaded from the GEO database using accession number GSE3678. The experiment profiles gene expression in 7 paired PTC patient samples and normal samples.

The third Affymetrix data set we used was generated by Oatley using Affymetrix mouse4302 chip [20]. GDNF-regulated gene expression was studied in cultures of actively self-renewing spermatogonial stem cells established from 6 day old male mice. GDNF is the essential growth factor regulating mouse spermatogonial stem cell self-renewal. The gene expression was measured prior to withdraw, after withdraw and 2, 4, 8 hours of GDNF/GFR*α *replacements with 3 replicates for each time points. The data was downloaded from the Gene Expression Omnibus (GEO) database using accession number GSE4799.

### The Algorithm

For genes with sibling probe sets, we fit the full two-way ANOVA model (Eq. 1) with probe set by treatment interaction to the pooled data. If the interaction effect *τψ *is insignificant after multiple-test correction (as we used *FDR *≤ 0.01, Benjamini-Hochberg Procedure [25]), we then report *P*-values of the treatment effect *τ*; otherwise we consider sibling probe sets as independent probe sets. For the gene corresponding to a single probe set and these independent probe sets, we fit the one-way ANOVA model (Eq. 3) where only model treatment effect is included.

#### Two-way ANOVA model

Let *y*_*ijk *_be the normalized and summarized probe set intensity score for the *i*_*th *_gene, *j*_*th *_probe set and *k*_*th *_replicates of this probe set, we model treatment effect (*τ*_*i*_), probe set effect *ψ*_*j *_and their interaction effect (*τψ*)_*ij *_as two factors with interaction having *i *and *j *levels, *i *= 1, 2, . . . , *I*, *j *= 1, 2, . . . , *J *where *I *represents the number of conditions to compare, and *J *represents the number of sibling probe sets for one gene:

(1) *y*_*ijk *_= *μ *+ *τ*_*i *_+ *ψ*_*j *_+ (*τψ*)_*ij *_+ *ε*_*ijk *_

Let *β *represents the block factor, where *k *presents block size, *k *= 1, 2, the two-way ANOVA model with block effect is:

(2) *y*_*ijk *_= *μ *+ *τ*_*i *_+ *ψ*_*j *_+ *β*_*k *_+ (*τψ*)_*ij *_+ *ε*_*ijk *_

#### One-way ANOVA model

Define *y*_*jk *_is the normalized and summarized probe set intensity score for *j*_*th *_probe set and *k*_*th *_replicates, we model treatment effect (*τ*_*j*_) as fixed effect having *j *levels, *j *= 1, 2, . . . , *I*:

(3) *y*_*jk *_= *μ *+ *τ*_*j *_+ *ε*_*jk *_

Similarly, the one-way ANOVA model with block effect is:

(4) *y*_*jk *_= *μ *+ *τ*_*j *_+ *β*_*k *_+ *ε*_*jk*_,

where *k *= 1, 2.

R function lm() was used to fit one-way and two-way ANOVA models.

### The Custom CDF Approach

Custom CDF files (version 8) were downloaded from [32] for hgu133plus2 and moe4302 platforms. Probe set definitions mapped to UniGene database, EntrezGene database and ensEMBL gene database were considered in this work. The probe set expression was calculated using one or all of three normalization methods (MBEI, RMA, GCRMA). The differentially expressed genes were identified using model 3 as were used for the per-probeset approach.

### GO Enrichment Analysis

For gene lists generated by per-gene or per-probeset approaches, we used Bioconductor package "GOstats" [33] to perform GO enrichment analysis. For gene lists generated by the customCDF approach, we retrieved counts of the GO terms that are associated with the differentially expressed gene list and the whole genome list by querying Ensemble databases, and then performed hypergeometric test using R function phyper.

## Authors' contributions

All authors read and approved the final manuscript. HL conceived and designed study. HL, DZ and MC analyzed data. HL and DZ drafted the manuscript.

## Supplementary Material

Additional file 1 **Excel file containing The list of significantly differentially expressed genes detected by the per-gene approach between wild type and Nrl knockout mice in example 1**. The genes highlighted in yellow are detected by both the per-gene and the per-probeset approach. The genes that are highlighted in red are detected by the per-gene approach only and are directed or indirected regulated by Nrl. Click here for file

Additional file 2 **Excel file containing comparison in terms of numbers of differentially expressed genes between paired samples using different multiple test correction procedures in Example 2**. Comparison of the per-gene approach, the per-probeset approach, the UniGene custom CDF approach, and the ensEMBL gene custom CDF approach in terms of screening differentially expressed genes between paired normal and tumor tissues in Example 2. Click here for file

Additional file 3 **Plots of gene expression profiles in two different experiments**. This figure shows the expression profiles of three genes in examples 1 and 3 on the same affymetrix platform (moe4302) discussed in this work. The first example studied differentially expressed genes between wild type and Nrl-knock out mouse. The third example studied GNDF-regulated gene expression changes over time. For the gene Arr3(the first row), the expression profiles for all 3 sibling probe sets are similar and sibling probe sets are consolidated in both experiments. For the gene Plxnc1 (the second row), the expression profiles in the first example are similar, but are dissimilar in the third example. Similarly for the gene HOD (the third row), the expression profiles for three sibling probe sets are very different in the first example, but are similar in the third example. So, it's possible that the sibling probe sets behave differently in different experiments that study different biological questions. The differences might represent annotation errors, unknown artifact, or alternatively spliced transcripts. Click here for file
